# Mucosal and cellular immune responses elicited by nasal and intramuscular inoculation with ASFV candidate immunogens

**DOI:** 10.3389/fimmu.2023.1200297

**Published:** 2023-09-01

**Authors:** Lulu Xu, Fei Hao, Dae Gwin Jeong, Rong Chen, Yuan Gan, Lei Zhang, Minjoo Yeom, Jong-Woo Lim, Yanfei Yu, Yun Bai, Zhiyong Zeng, Yongjie Liu, Qiyan Xiong, Guoqing Shao, Yuzi Wu, Zhixin Feng, Daesub Song, Xing Xie

**Affiliations:** ^1^ Joint International Research Laboratory of Animal Health and Food Safety, College of Veterinary Medicine, Nanjing Agricultural University, Nanjing, China; ^2^ Key Laboratory for Veterinary Bio-Product Engineering, Ministry of Agriculture and Rural Affairs, Institute of Veterinary Medicine, Jiangsu Academy of Agricultural Sciences, Nanjing, China; ^3^ GuoTai (Taizhou) Center of Technology Innovation for Veterinary Biologicals, Taizhou, China; ^4^ Jiangsu Key Laboratory for Food Quality and Safety-State Key Laboratory Cultivation Base, Ministry of Science and Technology, Nanjing, China; ^5^ Bionanotechnology Research Center, Korea Research Institute of Bioscience and Biotechnology, Daejeon, Republic of Korea; ^6^ College of Veterinary Medicine and Research Institute for Veterinary Science, Seoul National University, Seoul, Republic of Korea; ^7^ College of Animal Science, Guizhou University, Guiyang, China

**Keywords:** African swine fever virus (ASFV), ASFV-convalescent pig serum, mass spectrometry identification, recombinant protein expression, immunogenicity assessment

## Abstract

African swine fever (ASF) is an infectious disease caused by African swine fever virus (ASFV) that is highly contagious and has an extremely high mortality rate (infected by virulent strains) among domestic and wild pigs, causing huge economic losses to the pig industry globally. In this study, SDS−PAGE gel bands hybridized with ASFV whole virus protein combined with ASFV-convalescent and ASFV-positive pig serum were identified by mass spectrometry. Six antigens were detected by positive serum reaction bands, and eight antigens were detected in ASFV-convalescent serum. In combination with previous literature reports and proteins corresponding to MHC-II presenting peptides screened from ASFV-positive pig urine conducted in our lab, seven candidate antigens, including KP177R (p22), K78R (p10), CP204L (p30), E183L (p54), B602L (B602L), EP402R-N (CD2V-N) and F317L (F317L), were selected. Subunit-Group 1 was prepared by mixing above-mentioned seven ASFV recombinant proteins with MONTANIDETM1313 VG N mucosal adjuvant and immunizing pigs intranasally and intramuscularly. Subunit-Group 2 was prepared by mixing four ASFV recombinant proteins (p22, p54, CD2V-N1, B602L) with Montanide ISA 51 VG adjuvant and immunizing pigs by intramuscular injection. Anticoagulated whole blood, serum, and oral fluid were collected during immunization for flow cytometry, serum IgG as well as secretory sIgA antibody secretion, and cytokine expression testing to conduct a comprehensive immunogenicity assessment. Both immunogen groups can effectively stimulate the host to produce ideal humoral, mucosal, and cellular immune responses, providing a theoretical basis for subsequent functional studies, such as immunogens challenge protection and elucidation of the pathogenic mechanism of ASFV.

## 1 Introduction

African swine fever (ASF) is a serious swine infectious disease caused by African swine fever virus (ASFV), which can result in a mortality rate of nearly 100% infected by virulent strains (1). The main sources of ASF transmission in commercial pigs under artificial farming are contact with contaminated animal feed, pork or pig products, surfaces of contaminated objects and materials present in the environment, in addition to the rapid spread of the virus through body fluids and excretions of infectious animals in the enclosures (2, 3). A total of 24 ASFV genotypes have been identified based on the P72-encoding gene (B646L). ASF has been active in sub-Saharan Africa for most of the time since it was reported at the beginning of the last century. It was introduced to Georgia in 2007 and has been raging in Russia, Poland, Ukraine and other countries (4–6). After being introduced and the spread of the ASFV II genotype into China in 2018, it has rapidly spread in various provinces and cities in China, causing huge losses to the pig farming industry (7). Due to the complex protein structure and powerful immune evasion function of ASFV and the extremely rapid onset and short duration of the disease, the host often dies because the immune system does not have time to respond to virus invasion (8). Until now, there has been no effective vaccine for ASF in China and Korea, and the mainstream prevention and control approach is still to quarantine and cull suspected infected animals (9).

Conventional inactivated vaccines have proven to be ineffective in ASF control (10). At present, the research focuses are mainly on live virus-based natural attenuated live vaccines, gene deletion vaccines and antigen-based subunit vaccines, DNA vaccines, and virus vector vaccines (4). To date, the live attenuated vaccine (ASFV-G-ΔI177L) developed in Vietnam is the first commercially available African swine fever vaccine in the world (11). However, naturally attenuated strains generally have protective effects only on animals infected with homologous genotypes and have the risk of sustaining the infection and restoring virulence (12). Gene-deficient strains often have side effects such as swollen joints and varying degrees of viremia. In addition, the development of this vaccine has been plagued by the problem of stable cell lines (4, 13). In contrast, although antigen-based vaccines are restricted by factors such as antigen screening, immunization strategy and adjuvant selection, there is no doubt that they provide a safer method with fewer side effects. Many antigens from ASFV have been tested as subunit vaccines, including p30, p54, CD2v and other proteins (14–16). They were capable of delaying the onset of clinical symptoms, reducing viremia and inducing at least a minimal immune response (17, 18). Although none of the antigens successfully protected the host against a virulent virus challenge, they could still be considered to be potential antigens for future vaccine development.

The serum of ASFV-convalescent pigs were collected from domestic pigs or wild boars infected with ASFV and recovered under non-artificial conditions. The serum of ASFV-positive pigs were collected from pigs infected with ASFV and died. ASFV-negative pig serum were collected from healthy pigs that had never been infected with ASFV. ASFV-convalescent pigs are domestic or wild pigs that have been infected with ASFV and recovered under nonartificial conditions. We presume that the serum of ASFV-convalescent pigs contains critical ASFV protective antibodies. Thus, our research firstly through immunological hybridization of convalescent pig serum with ASFV whole virus proteins and subsequent mass spectrometric analysis, to identify ASFV antigens capable of inducing protection, immunogens of which may be subunit vaccine candidates. In addition to screening for candidate immunogens, the immunization route is another important factor in the effectiveness of subunit vaccines (4). Moreover, we used prokaryotic and eukaryotic protein expression systems to express recombinant ASFV proteins. According to our previous study, a combination of intranasal and intramuscular immunization routes can be effective in stimulating host to produce a more robust immune response by using subunit vaccines containing MONTANIDE™1313 VG N adjuvant (19).

Therefore, in this study, immunogens of two subunit vaccine candidates were identified by mass spectrometry through inactivated ASFV-convalescent serum (provided by Guizhou University) and inactivated ASFV-positive serum (provided by Harbin Veterinary Research Institute, CAAS) immunoblotting with ASFV whole virus proteins. Two adjuvant formulations and two different immunization routes were selected to prepare ASFV immunogen group-1, named Immunogen Group 1 and immunogen group-2 named Immunogen Group 2, respectively. Anticoagulated whole blood, serum, and oral fluid samples were collected during immunization for flow cytometry, serum IgG as well as secretory sIgA antibody and cytokine expression levels for conducting a comprehensive immunogenicity assessment of immunogen candidates in the primary immunization and booster programs.

## 2 Materials and methods

### 2.1 Ethics approval

Animal experiments were approved by the Committee on the Ethics of Animal Experiments (Protocol # PDC 2022013) and were performed in strict accordance with the animal regulations of Jiangsu Province (Government Decree No. 45) at Jiangsu Academy of Agricultural Sciences (License No. SYXK (Su) 2020-0023).

### 2.2 Immunoblot assay with ASFV-convalescent pig serum

Purified ASFV-whole virus protein samples (inactivated, provided by Harbin Veterinary Research Institute, CAAS) were mixed with 5× SDS loading buffer at a ratio of 4:1. All samples were heated for 10 min at 100°C, spun down, and separated under 10% separating SDS−PAGE gels (E303-01, Vazyme, Nanjing, China). The electrophoresis was run in Tris-glycine buffer at pH 8.0 and 90 V for 15 min before at 120 V until the sample dye reached the end of the gel. The gels were stained with 0.2% Cmassie Brilliant Blue R-250 and unstained for 10 min at 100°C water.

For Western blot analysis, the detailed protocol followed a previous study with some modifications (20). Briefly, the electrophoresed proteins were transferred to a polyvinylidene difluoride membrane using a semidry transfer apparatus (Bio-Rad) for 30 min at a constant voltage of 20 V. The membrane was rinsed twice with 25 mM Tris-HCl buffer at pH 8.0 containing 0.9% NaCl and 0.05% Tween-20 (Tris-buffered saline named TBST), blocked with TBST containing 5% skimmed milk and rinsed the membrane three times for 5 min in TBST, and then incubated with a 1:200 diluted ASFV- convalescent pig serum 2 h in 37°C, and rinsed the membrane three times for 5 min. Immunodetection was performed for 2 h with a 1: 10, 000 diluted HRP-conjugated goat anti-pig IgG (Cat No. SA00001-1, Chicago, USA). Finally, the membranes were developed with Electro-ChemiLuminescence (ECL) substrate (Cat No. 180-5001, Tanon, Shanghai, China) using a ChemiDoc XRS+ system (Bio-Rad, Hercules, CA, USA). As a molecular mass marker, Novex Sharp Pre-Stained Protein Standard (Cat No. 26628, Thermo Fisher Scientific, NY, USA) was used.

### 2.3 Protein shotgun identification analysis

Mass spectrometric identification of gel bands from blotting analysis was performed according to the instructions of Shanghai Biospectrum Biotechnology Co. The detailed procedure was as follows (21). Briefly, the SDS−PAGE gels were decolorized by Komas Brilliant Blue staining, and the bands that produced specific binding were cut. The stained gel block samples were subsequently subjected to proteolytic digestion. The enzymatically digested samples were subjected to LC−MS/MS analysis using a chromatography system (Thermo Scientific). Mass spectrometry database search software MaxQuant 2.0.1.0. and the following protein database: NCBI-asfarviridae [137992]-46414-20221202.fasta (https://www.ncbi.nlm.nih.gov/protein/? term=Asfarviridae); UniProt - Asfarviridae[137992]-8024-20221202.fasta (https://www.uniprot.org/uniprotkb? Query=(taxonomy_id:137992)) was used. The Mascot parameters for all databases are listed in **Table 1**.

**Table 1:** Parameters of mass spectrometry identification.

Item	Value
**Enzyme**	Trypsin
**Max Missed Cleavages**	2
**Precursor Tolerance (Main search)**	4.5 ppm
**Precursor Tolerance (First search)**	20 ppm
**MS/MS Tolerance**	20 ppm
**Fixed modifications**	Carbamidomethyl (C)
**Variable modifications**	Oxidation (M), Acetyl (Protein N-term)
**Database**	ncbi-Asfarviridae [137992]-46414-20221202.fasta; UniProt-Asfarviridae [137992]-8024-20221202.fasta
**Database pattern**	Target-Reverse
**PSM FDR**	0.01
**Protein FDR**	0.01
**Site FDR**	0.01

### 2.4 Construction and expression of recombinant proteins

The gene sequences of KP177R, K78R, CP204L, E183L, B602L, EP402R-N, and F317L of the ASFV China/2018/PIG/HLJ strain with the GenBank accession number MK333180.1 were codon optimized using codon optimization software (http://www.jcat.de/) for *Escherichia coli* (*E. Coli*) expression. The optimized gene sequences were synthesized by Nanjing GenScript Biotechnology Co., Ltd. For Immunogen Group 1, KP177R and E183L were cloned into the pET-21a vector, and K78R, CP204L, B602L, and EP402R-N were cloned into the pET-32a vector, and F317L was cloned into the pFastBac vector. ASFV recombinant proteins were then expressed in the BL21(DE3) *E. coli* strain and baculovirus system. For Immunogen Group 2, the recombinant p22 (pAcGP67a-KP177R-6His), CD2V-N1 (pAcGP67a-EP420R-6His) proteins (expressed in baculovirus system), and p54 (pET28a-E183L), B602L (pET28a-B602L) proteins (expressed in the *E. coli)* were provided by Korea Research Institute of Bioscience and Biotechnology (KRIBB). The bacteria were grown in LB medium at 37°C until the OD600 reached approximately 0.8. A final concentration of 0.4 mM IPTG was added to the culture for protein expression at 18°C for 22 h at 120 rpm/min, bacteria were collected for ultrasonic crushing, and the supernatant was used for protein purification. The supernatant of stably transfected Sf9 cells was collected for protein purification. The soluble fraction was incubated with Ni Sepharose 6 FF resin (GE Healthcare, USA) for 1 h at 4°C. Proteins were eluted in buffer A by adding imidazole and concentrated by ultrafiltration using Centricons (Cat No. UFC9010, Millipore, USA). Final concentrations of the proteins were dissolved in Buffer A and expressed as milligrams of protein per liter of bacterial solution determined with the BCA Protein Assay Kit (Cat No. P0012S, Beyotime, Shanghai, China) before storage at -70°C.

### 2.5 Immunoblot assay with pig ASFV-positive and ASFV- convalescent serum

The purified ASFV recombinant protein samples were separated on 10% separating SDS−PAGE gels. The electrophoresed proteins were transferred to a polyvinylidene difluoride membrane. The membrane prepared as mentioned above was cut into strips, blocked with TBST containing 5% skimmed milk, and incubated for 2 h with 1:200 diluted pig ASFV-positive or ASFV-convalescent serum at 37°C. The rest of the method is the same as mentioned above.

### 2.6 Preparation of polyclonal antibodies and immunoblot assay

For preparation of polyclonal antibodies, 2 mL of purified ASFV recombinant proteins p22 and p10 (1 mg/mL) was emulsified with an equal volume of Freund’s complete adjuvant (Cat No. F5881, Sigma−Aldrich, St Louis, MO, USA) in the first immunization wherein 4.00 mL was injected subcutaneously into New Zealand rabbits. From the second to third immunization, Freund’s complete adjuvant was replaced with Freund’s incomplete adjuvant (Cat No. F5506, Sigma−Aldrich, St Louis, MO, USA). All immunizations were carried out at two-week intervals. Blood samples were collected two weeks after the last immunization.

The membrane prepared of proteins p22 and p10 as described above was cut into strips, blocked with TBST containing 5% skimmed milk, and incubated for 2 h with 1:3000 diluted rabbit serum at 37°C. The rest of the method is the same as mentioned above.

### 2.7 Mass expression of recombinant ASFV proteins and immunogen candidates preparation

For ASFV immunogen group-1 named Immunogen-Group (G) 1 was prepared by mixing seven ASF recombinant proteins (KP177R (p22), K78R (p10), B602L, EP402R-N (CD2V-N), F317L, CP204L (p30) and E183L (p54), each protein 0.1 mg diluted totally in 1 mL PBS), according to the volume ratio of 1: 1 mixed with MONTANIDE™1313 VG N (SEPPIC, Puteaux, France) mucosal adjuvant and immunizing pigs by a combination of intranasally (IN) and intramuscularly (IM) with 2 mL/piglet (1 mL IM & 1 mL IN) once immunization.

Similarly, for ASFV immunogen group-2 named Immunogen Group-2, which was prepared by mixing four ASF recombinant proteins (p22 (0.155 mg), p54 (0.15 mg), CD2V-N1 (0.145 mg), B602L (0.12 mg) from KRIBB diluted totally in 0.75 mL PBS) together with 0.75 mL Montanide ISA 51 VG adjuvant and immunizing pigs by intramuscular injection with 1.5 mL/piglet once immunization. For one immunization, 4.5 mL of four purified ASFV recombinant proteins were mixed with an equal volume of 51 adjuvant, and ASFV Immunogen*-*G2 was obtained.

### 2.8 Experimental animals, immunization and sample collection

Thirteen healthy piglets (four-week-old male, large Yorkshire) with neither signs of cardio-pulmonary disorders nor under any drug treatment or vaccination were purchased from Nanjing Zhoubang Bio-Tech Co., Ltd. Before conducting the pig experiment, an antibody test based on serological analysis showed that all the following pathogens were negative, including porcine respiratory and reproductive syndrome virus (PRRSV), pseudorabies virus (PRV), ASFV, classical swine fever virus (CSFV) and porcine circovirus type 2 (PCV2). To evaluate the immunogenicity of the ASFV candidate immunogens, 13 piglets were randomly divided into three groups, two experimental groups (5 piglets/group) and one control group containing three piglets, and the experimental scheme is shown in **Table 2**. Each piglet in the experimental Immunogen-Group 1 was immunized with 2 mL of the above prepared vaccine intranasally and intramuscularly, and each immunization method used 1 mL/piglet. Every piglet in the experimental Immunogen-Group 2 was intramuscularly immunized with 1.5 mL of the above prepared vaccine. The negative control group was immunized intramuscularly with 1 mL phosphate-buffered saline (PBS) and intranasally with 1 mL PBS. In two weeks, piglets were again boosted with the same dose and route of vaccines or PBS as described.

**Table 2:** Experimental groups and administration strategies.

group	number	immunogen	0 d	14 d
**G1**	5	ASFV-p22, p10, p30, p54, B602L, CD2V-N and F317L mixed with MONTANIDETM1313 VG N	IN+IM	IN+IM
**G2**	5	ASFV- p22, p54, CD2V-N1 and B602L mixed with Montanide ISA 51 VG adjuvant	IM	IM
**G3**	3	PBS	IN+IM	IN+IM

IM, intramuscular inoculation; IN, intratumoral inoculation; IM+IN, intratumoral and intramuscular inoculation.

At 0, 7, 14, and 28 days post immunization (dpi), 100 μL of EDTA anticoagulated whole blood, 2 mL of nonanticoagulated blood samples and 1 mL of oral fluid were collected, anticoagulated whole blood was stored at 4°C for flow cytometry detection, supernatant serum and oral fluid were obtained and frozen at -20°C after centrifugation at 4,000 × *g* for 10 min at 4°C for IgG antibody and cytokine expression detection, and oral fluid was used for sIgA antibody detection. At 28 dpi, pigs were euthanized and necropsy.

### 2.9 Flow cytometry analysis

Anticoagulated peripheral blood (100 μL) was stained with the pig monoclonal antibodies CD3-FITC (Cat. No MA5-41029), CD4-PE (Cat. No MA5-28733), and CD8-APC (Cat. No MA5-28712) (0.1 mg/mL/test, all from Invitrogen, NY, USA) for 25 min at room temperature (RT) in separate 1.5 mL EP tubes, and single-labeled groups with three individually labeled antibodies were set up simultaneously. Two microliters (10 times dilution with PBS) of red blood cell lysis buffer (Cat No. QYR042, Fcmacs, Nanjing, China) was subsequently added, and the cells were well mixed gently by flicking the bottom of the tube. The tubes were kept at RT for 15–30 min in the dark until the liquid became clear and transparent before being centrifuged at 4°C for 5 min at 1000 rpm. The pellet was resuspended in 1 mL of PBS, and the cells were resuspended in 200 μL of PBS after two PBS washes. Then, 20 μL of 4% paraformaldehyde was added to the final suspension, and the tubes were placed at 4°C overnight one day before use. A total of 2 × 10^4^ lymphocytes were analyzed with a BD flow cytometer (BD FACSCalibur, NY, USA). The results are expressed as percentages of CD3^+^ CD4^+^ T cells as well as CD3^+^ CD8^+^ T cells in the total lymphocyte population in five replicates.

### 2.10 Indirect enzyme-linked immunosorbent assay for specific serum IgG and oral fluid sIgA antibody detection

The specific serum IgG and oral fluid sIgA antibodies of the above two ASFV subunit vaccine candidates were detected by indirect ELISA. Specifically, 96-well plates (Corning, Shanghai, China) were coated with KP177R (p22), K78R (p10), B602L (B602L), EP402R-N (CD2V-N), F317L (F317L), CP204L (p30) and E183L (p54) purified protein (2 μg/well, diluted in PBS) and ASFV whole virus (5 μg/well, diluted in PBS) for detection of serum IgG, and 96-well plates (Corning, Shanghai, China) were coated with p22 plus p30 (2 μg/protein/well) and ASFV whole virus (5 μg/well, diluted in PBS) for detection of oral fluid sIgA. After overnight incubation at 4°C, the plates were washed three times with PBS containing 0.1% Tween 20 (PBST) and blocked for 2 h at RT with 200 μL of PBST plus 5% skimmed milk. One hundred microliters of sera (1:100 dilution in PBST) or oral fluid (no dilution) was added. The plates were incubated for 2 h at 37°C, and detection of bound immunoglobulins was performed by adding 100 μL of HRP-conjugated goat anti-pig IgG (1:20,000) (Cat No. SA00001-1, Chicago, USA) or HRP-conjugated goat anti-pig IgA (1:10,000) (Cat. No A90-103P, BETHYL, Shanghai, China). TMB solution (100 μL/well, Cat No. P0209, Beyotime, Shanghai, China) was added after three washes. After 10 min of incubation in the dark at 37°C, the reaction was stopped by adding 50 μL of 2 mol/L H_2_SO_4_ before measuring the optical density (OD) at 450 nm (IgG or sIgA), expressed as OD values.

### 2.11 Real-time quantitative polymerase chain reaction and ELISA for pig cytokine expression detection

Total RNA from each serum sample was extracted according to the manufacturer’s instructions (LOT: 05921KC5, Axygen, NY, USA). Briefly, 1 μg of total RNA was reverse transcribed in a 10 µL reaction volume using HiScript® III RT SuperMix for qPCR (+gDNA wiper) (Cat No. R323-01, Vazyme, Nanjing, China) before running on an ABI 7500 Real Time PCR System using the ChamQ SYBR qPCR Master Mix (Cat No. Q311, Vazyme, Nanjing, China). The qPCR assay was performed using cDNA under specific conditions with the following procedure: Stage 1: 95°C for 30 s; Stage 2: (10 s at 95°C, 30 s at 60°C) was repeated for 40 cycles; Stage 3: 95°C for 15 s, 60°C for 60 s, 95°C for 15 s. The β-Actin gene was used as the internal control. The PCR primers used in the quantitative assays are listed in **Table 3**. The fold change in pig mRNA expression of cytokine genes including Interleukin (IL)-2, IL-6, IL-10, Interferon (IFN)-α, IFN-γ, Tumor necrosis factor (TNF)-α between immunization groups and the negative control group was determined using the 2^-ΔΔCT^ method (22). At the same time, according to the standard curve drawn by each cytokine standard curve, and the (OD450-OD630) value, the six cytokines including IL-2 (Cat.No SEKP-0002), IL-6 (Cat.No SEKP-0004), TNF-α (Cat.No SEKP-0009), IL-10 (Cat.No SEKP-0007), IFN-α (Cat.No SEKP-0010), IFN-γ (Cat.No SEKP-0045), all purchased from Solarbio Bio. (Beijing, China), and concentrations of cytokines from each pig serum of two subunit vaccine groups were calculated.

**Table 3:** Pig cytokine primer information for qRT−PCR analysis.

Molecule	Sense Primer (5’-3’)	Anti-sense Primer (5’-3’)
TNF-α	CCAATGGCAGAGTGGGTATG	TGAAGAGGACCTGGGAGTAG
IFN-α	GGCTCTGGTGCATGAGATGC	CAGCCAGGATGGAGTCCTCC
IFN-γ	GCTCTGGGAAACTGAATGAC	TCTCTGGCCTTGGAACATAG
IL-6	ATCAGGAGACCTGCTTGATG	TGGTGGCTTTGTCTGGATTC
IL-10	GCATCCACTTCCCAACCA	CTTCCTCATCTTCATCGTCAT
IL-2	AAGCACAGCAGCAGCAGCAG	GCCGCAGAGGTCCAAGTTCATC
β-actin	CATCACCATCGGCAACGA	GCGTAGAGGTCCTTCCTGATGT

### 2.12 Statistical analysis

All data came from at least three independent experiments and are expressed as the mean ± SD. GraphPad Prism 8.3 software was used to compare the differences. Two-way analysis of variance (ANOVA) followed by Tukey’s multiple-comparison test for *post hoc* analysis. *P* < 0.05 was considered to indicate a significant difference, and *P* < 0.01 and *P* < 0.001 were considered to indicate an extremely significant difference.

## 3 Results

### 3.1 Protein identification from ASFV-convalescent and ASFV-positive pig serum

**Figure 1:**
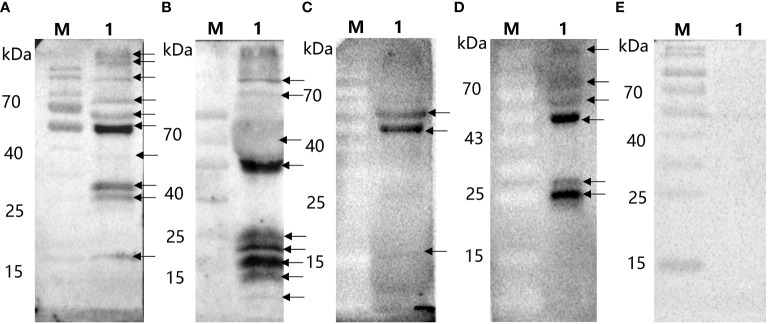
Immunoblotting of whole virus protein against ASFV-convalescent pig serum. **(A)** ASFV-convalescent pig serum I as a primary antibody: M: Protein marker; 1: ASFV whole virus protein. **(B)** ASFV-convalescent pig serum II as a primary antibody: M: Protein marker; 1: ASFV whole virus protein. **(C)** ASFV-positive pig serum I as a primary antibody: M: Protein marker; 1: ASFV whole virus protein. **(D)** ASFV-positive pig serum II as a primary antibody: M: Protein marker; 1: ASFV whole virus protein. **(E)** ASFV-negative pig serum as a primary antibody: M: Protein marker; 1: ASFV whole virus protein.

After the ASFV whole virus protein reacted with ASFV-positive and ASFV-convalescent serum, as shown in **Figure 1**, the results showed that after positive and convalescent pig serum reacted with the whole ASFV virus protein, corresponding proteins were identified, and there were differences between positive pig serum and convalescent pig serum.

### 3.2 Mass spectrometry identification and determination of ASFV candidate immunogens

**Figure 2:**
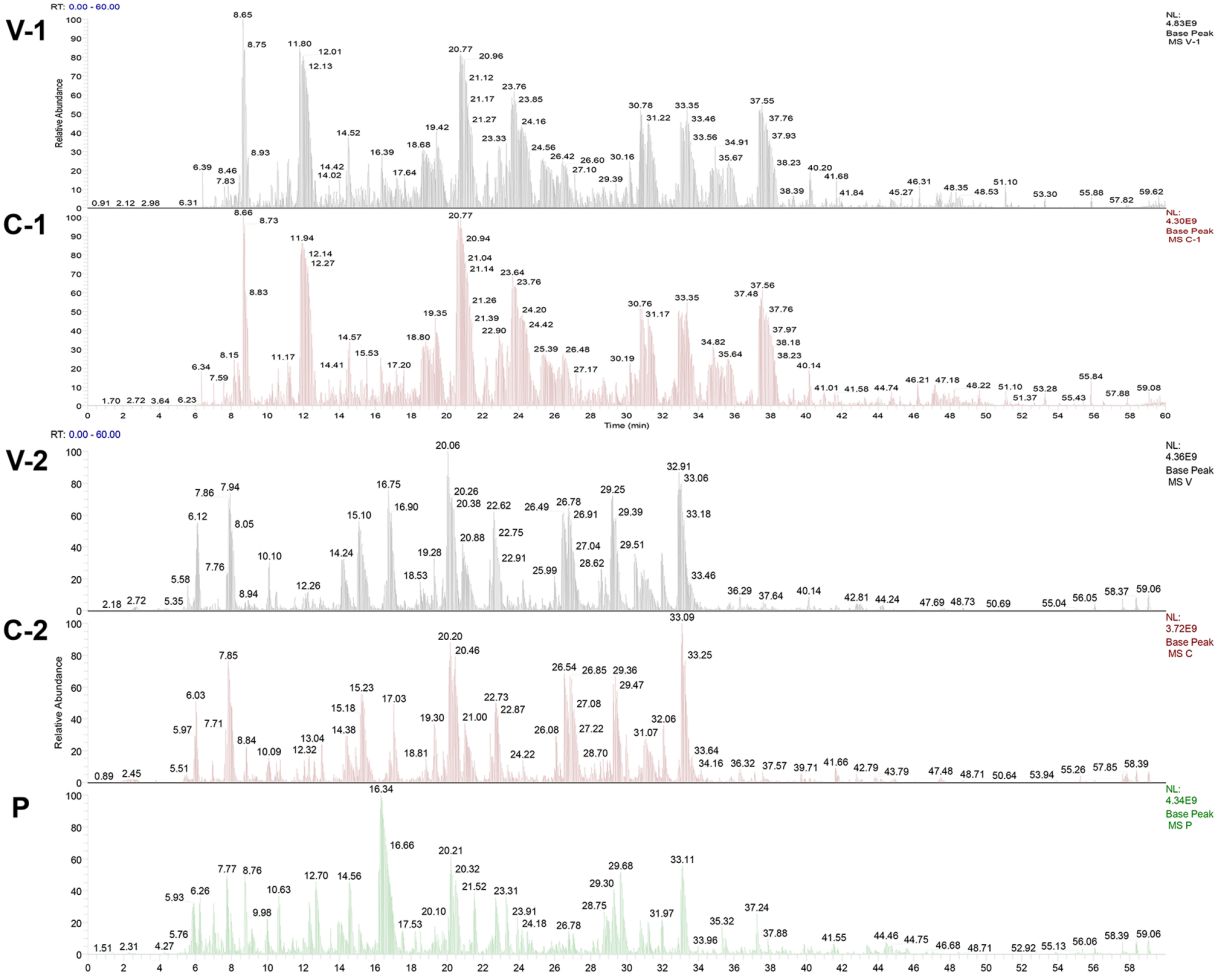
Mass spectrometry identification peak map of ASFV-specific bands. V-1 and V-2: Mass spectrometry identification peak map against ASFV whole virus; C-1 and C-2: Mass spectrometry identification peak map against ASFV-convalescent pig serum; P: Mass spectrometry identification peak map against ASFV-positive pig serum.

After Coomassie brilliant blue staining on the SDS−PAGE gel of ASFV whole virus protein, the identified bands were cut off and sent to the sample for protein qualitative mass spectrometry detection (the results are shown in **Figure 2**), and the protein screened by the ASFV-convalescent pig serum, especially proteins screened by the ASFV-convalescent pig serum, was subtracted from ASFV-positive proteins. Eight antigens were detected by ASFV-convalescent pig serum reaction bands, and six antigens were detected by ASFV-positive pig serum bands. Among them, four of the proteins reacted with both positive and convalescent sera, as shown in **Table 4**.

**Table 4:** ASFV antigen screened by mass spectrometry identification.

Gel band	Antigen
**V**	CP2475, E165R, F1055L, E184L, M448R, R298L, MGF 360-8 L, MGF 110-11 L-10 L, B646L, CP312R, MGF-505-6R, CP204L, K205R, H339R, E423R, B475L, MGF 505-4R
**C**	E165R, F1055L, E184L, M448R, CP312R, CP204L, K205R, B475L
**P**	CP2475L, E165R, F1055L, E184L, M448R, R298L

V: ASFV whole virus protein; C: ASFV proteins against ASFV-convalescent pig serum; P ASFV proteins against ASFV-positive pig serum.

According to the results of mass spectrometry identification of the above convalescent pig serum and positive pig serum, combined with previous literature reports (16), as well as the screening of MHC-II presenting peptides from infected pig urine conducted in our lab (data not shown), the following seven antigens KP177R (p22), K78R (p10), CP204L (p30), E183L (p54), B602L (B602L), EP402R-N (CD2V-N), and F317L (F317L) were selected as candidate immunogens.

### 3.3 Large expression and purification of candidate immunogens

**Figure 3:**
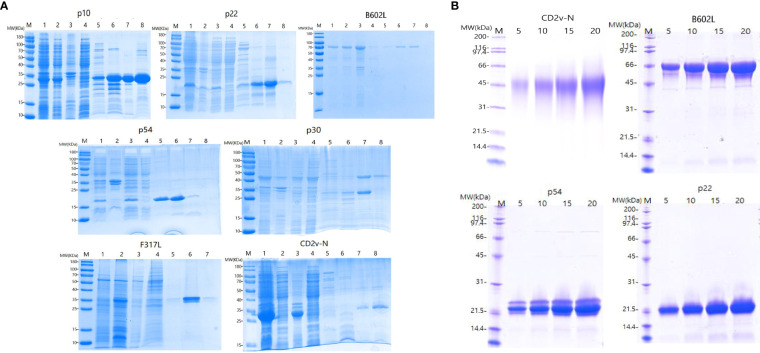
Expression and purification of ASFV recombinant proteins. **(A)** Purification and SDS−PAGE of the proteins p10, p22, B602L, p54, p30, F317L and CD2V-N expressed by *E Coli* prokaryotic expression system and baculovirus system; **(B)** Purified and SDS−PAGE of CD2V-N, B602L, p54 and p22 proteins expressed by *E coli* prokaryotic expression system and baculovirus system from KRIBB; Picture annotation of prokaryotically expressed p22, B602L, p54, p30, CD2V-N proteins: M: Protein marker; 1: Induced whole bacterial protein; 2: Bacterial precipitation after ultrasonic crushing; 3: Supernatant of bacteria after ultrasonic crushing; 4: Clear liquid on bacteria passes through liquid; 5-8: Purified proteins at imidazole by 50/100/250/500 mmol/L and refolded by PBS. Picture annotation of prokaryotically expressed p10 protein: M: Protein marker; 1: Induced whole bacterial protein; 2: Supernatant of bacteria after ultrasonic crushing; 3: Bacterial precipitation after ultrasonic crushing; 4: Clear liquid on bacteria passes through liquid; 5-8: Purified proteins at imidazole by 50/100/250/500 mmol/L and refolded by PBS. Picture annotation of baculovirus expressed F317 protein: M: Protein marker; 1: Induced whole virus protein; 2: Supernatant of bacteria after ultrasonic crushing; 3: Bacterial precipitation after ultrasonic crushing; 4-7: Purified proteins at imidazole by 50/100/250/500 mmol/L and refolded by PBS. Picture annotation of proteins expressed in prokaryotic expression system and baculovirus system from KRIBB M: Protein marker; 5, 10,15, 20 represents different loadings for each protein.

As shown in **Figure 3**, the SDS−PAGE results showed that the p22, p10, p30, p54, B602L, CD2V-N, and F317L proteins were successfully expressed in the prokaryotic expression system of *E. coli* and baculovirus system (**Figure 3A**), and the p22, p54, CD2V-N1, and B602L proteins were successfully expressed in the *E. coli* and baculovirus system from KRIBB (**Figure 3B**). As shown in **Figure 3A**, the molecular weights of the seven proteins p22, p10, p30, p54, B602L, CD2v-N, and F317L were 20.2 kDa, 30.4 kDa, 45.0 kDa, 19.9 kDa, 68.1 kDa, 35.7 kDa, 37.8 kDa, respectively. As shown in **Figure 3B**, the molecular weights of four proteins, p22, p54, B602L and CD2v-N1 were 20.07 kDa, 19.9 kDa, 65.11 kDa and 46.5 kDa. Because of the existence of glycosylation sites, the real size of proteins were bigger than the calculated amino acid size.

### 3.4 Different reactions from ASFV-positive and ASFV-convalescent sera against whole virus

**Figure 4:**
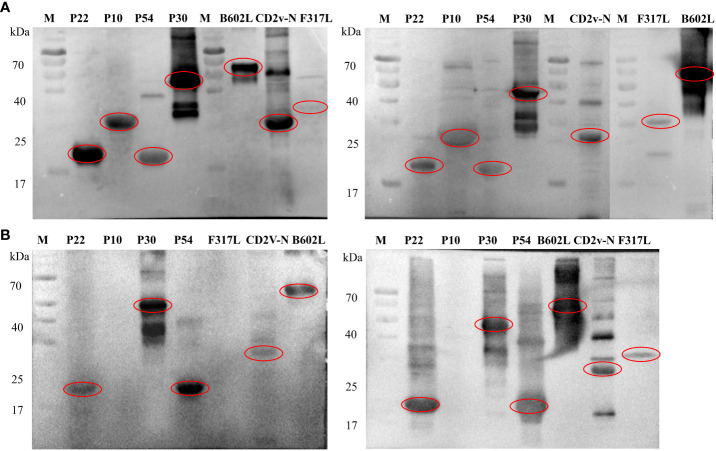
Immunoblot assay against pig ASFV-positive and ASFV-convalescent serum. **(A)** The first antibody is the immunoblot identification map of ASFV-positive pig serum. M: Protein marker, followed by the name of recombinant proteins. **(B)** The first antibody is the immunoblot identification map of ASFV-convalescent pig serum. M: Protein marker, followed by the name of recombinant proteins.

Western blot analysis showed that all the recombinant proteins could have immune binding with the serum from ASFV-positive pigs, and F317L had weak binding with positive serum, as shown from the two pig ASFV-positive serum samples, which was the same for mass spectrometry identification (**Figure 4A**). For the result of whole ASFV virus with ASFV-convalescent pig serum, it can be observed that p22, p30, p54, B602L and CD2V-N proteins have strong specific binding with both convalescent sera, p10 protein could not bind with convalescent serum, and F317L could bind with one of the ASFV-convalescent pig sera (**Figure 4B**).

### 3.5 Preparation of polyclonal antibodies and immunoblot assay

**Figure 5:**
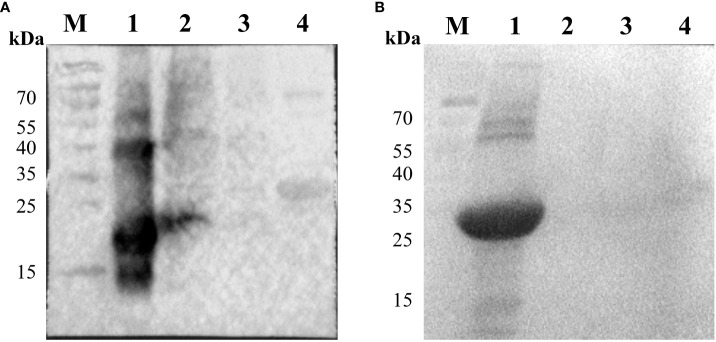
Identification of P22 and P10 recombinant proteins by Western blot with rabbit polyclonal antibody. **(A)** The first antibody is the serum of New Zealand white rabbits immunized with the p22 protein. M: Protein marker; 1: Protein p22; 2: Porcine alveolar macrophages (PAM) infected by ASFV (inactivated); 3: Normal PAM; 4: Unrelated protein p10. **(B)** The first antibody is the serum of New Zealand white rabbits immunized with the p10 protein. M: Protein marker; 1: Protein p10; 2: Porcine alveolar macrophages (PAM) infected by ASFV (inactivated); 3: Normal PAM; 4: Unrelated protein p54.

The prepared rabbit polyclonal antibodies against p22 and p10 were identified with the p22 and p10 proteins as primary antibodies. As shown in **Figure 5**, the p22 rabbit polyclonal antibody reacted with both the p22 protein and ASFV whole virus, while the p10 rabbit polyclonal antibody reacted with the p10 protein but did not bind to ASFV whole virus.

### 3.6 Mass expression of recombinant proteins and ASFV candidate immunogens preparation

Large amounts of proteins were expressed, the expression system of each pronucleus protein was expanded to 4 L, and the baculovirus system was expanded three times. For Immunogen-Group 1, prokaryotic proteins p22 (120 mg), p10 (150 mg), p30 (70 mg), p54 (80 mg), B602L (30 mg), CD2V-N (25 mg), and eukaryotic protein F317L (4 mg) were obtained. For Immunogen-Group 2, eukaryotic proteins p22 (50 mg), p54 (40 mg) and prokaryotic proteins B602L (50 mg) and CD2v-N (30 mg) were obtained.

### 3.7 CD4^+^ T-cell immunity was significantly activated after immunization

**Figure 6:**
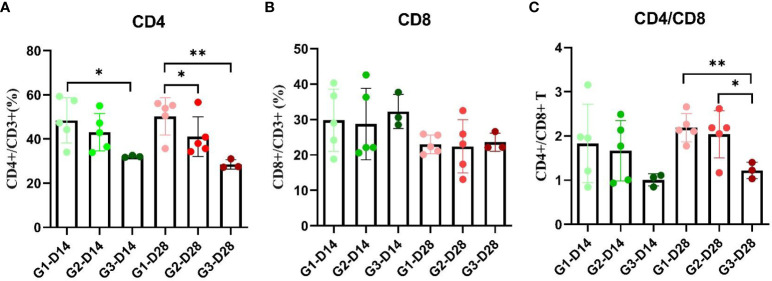
T lymphocyte cell grouping of animals immunized with two combination of candidate immunogens. Anticoagulated peripheral blood (100 μL) from each piglet of two subunit vaccine groups and negative control group at 14 and 28 dpi was stained with the pig monoclonal antibodies CD3-FITC, CD4-PE, and CD8-APC (0.1 mg/mL/test) for 25 min at RT in separate 1.5 mL EP tubes, and single-labeled groups with three individually labeled antibodies were set up simultaneously. **(A)** Percentage of CD4^+^ and CD3^+^ T cells at 14 dpi and 28 dpi in PBMCs of the G1 and G2 immune groups. **(B)** Percentage of CD8^+^ and CD3^+^ T cells at 14 dpi and 28 dpi in PBMCs of the G1 and G2 immune groups. **(C)** Percentage of CD4^+^ and CD8^+^ T cells at 14 dpi and 28 dpi in PBMCs of the G1 and G2 immune groups. Data reflect the means ± SDss of three independent experiments. *: *p* < 0.05 indicates that the difference is significant, while **: *p* < 0.01 indicates that the difference is extremely significant.

Compared with the negative control group, the percentage of CD4^+^/CD3^+^ cells in peripheral blood mononuclear cells (PBMCs) of the ASFV Immunogen-Group 1 was significantly increased after both one immunization for two weeks (*p <*0.05, **Figure 6A**) and a second immunization at 28 dpi (*p <*0.05, **Figure 6A**). Two weeks after the second immunization, the percentage of CD4^+^/CD3^+^ cells in PBMCs in Immunogen-Group 1 was also significantly increased compared with that in Immunogen-Group 2 (*p <*0.05, **Figure 6A**). Compared to the negative control group, the percentage of CD8+/CD3+ lymphocytes had no significant differences after the first or second immunization of both immunogen groups (**Figure 6B**). The CD4+/CD8+ T-cell ratio in PBMCs of Immunogen-Group 1 (*p <*0.01) and Immunogen-Group 2 (*p <*0.05) was significantly increased two weeks after the second immunization, and the Immunogen-Group 2 was relatively weak (**Figure 6C**). The results showed that both immunization groups could both strengthen CD4^+^ T-cell immunity two weeks after the second immunization, which made the host produce an immune response biased toward CD4^+^ T cells.

### 3.8 Serum and mucosal antibodies were significantly activated after immunization

**Figure 7:**
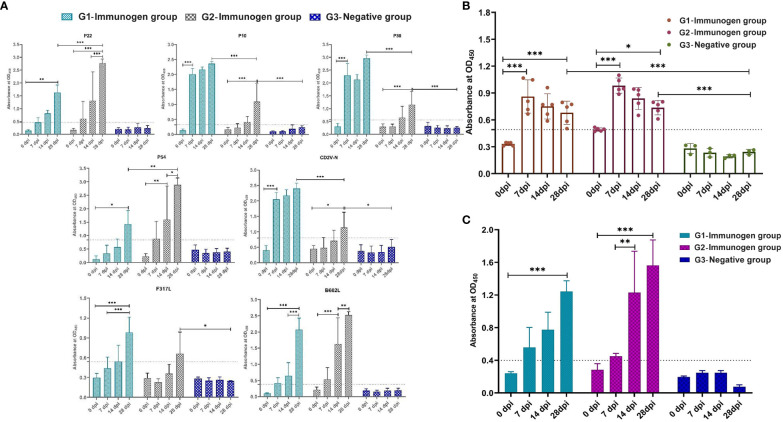
Pigs immunized with two combination of candidate immunogens induce high levels of IgG and sIgA antibodies. The specific serum IgG and oral fluid sIgA antibodies of the above two ASFV subunit vaccine candidates were detected by indirect ELISA. 96-well plates were coated with KP177R (p22), K78R (p10), B602L (B602L), EP402R-N (CD2V-N), F317L (F317L), CP204L (p30) and E183L (p54) purified protein (2 μg/well, diluted in PBS) and ASFV whole virus (5 μg/well, diluted in PBS) for detection of serum IgG, and 96-well plates (Corning, Shanghai, China) were coated with p22 plus p30 (2 μg/protein/well) and ASFV whole virus (5 μg/well, diluted in PBS) for detection of oral fluid sIgA. **(A)** One hundred-fold diluted serum-specific IgG antibody expression levels in candidate immunogen immunized pigs coated with recombinant ASFV proteins. **(B)** Tenfold diluted serum-specific IgG antibody expression levels in candidate immunogen-immunized pigs coated with ASFV whole virus protein. **(C)** Oral fluid-specific sIgA antibody expression levels in candidate immunogen-immunized pigs with double-coated proteins p22 plus p30. Data reflect the means ± SDss of three independent experiments. *: *p* < 0.05 indicates that the difference is significant, **: *p* < 0.01, ***: *p* < 0.001 indicates that the difference is extremely significant. The dashed line represents the critical value of iELISA for specific serum IgG and oral fluid sIgA antibody, which is 2.1 times that of negative serum OD450.

The purified protein was used to detect the IgG antibody in the serum of animals immunized at 0, 7, 14 and 28 dpi. As shown in **Figure 7A**, the secretion of IgG antibody in the serum of p22 protein in both G1 and G2 group increased gradually within 28 day period, and serum IgG expression level in G1 at 28 dpi increased significantly compared to that at 0 (G1 and G2) and 14 dpi (G1) (*p* <0.01, *p* <0.001), and the secretion of serum IgG antibody of p22 protein in G2 group was significantly higher than that in G1 group at 28 dpi, indicating that the secretion of serum IgG antibody of p22 protein in G2 group increased significantly in the period of 14-28 dpi (*p* <0.01, *p* <0.001). The secretion of serum IgG antibodies against p10, p30 and CD2V-N in the G1 group increased rapidly within 7 dpi and gradually increased from 7 to 28 dpi, which was significantly higher than that at 0 dpi (*p <*0.001). The secretion of serum IgG antibodies against p10, p30 and CD2V-N in the G2 group increased rapidly from 0 to 28 dpi (*p <*0.001, CD2V-N for *p* < 0.05), and the serum IgG antibodies against p10, p30 and CD2V-N in the G2 group were significantly lower than those in the G1 group at 28 dpi (*p <*0.001). The secretion of serum IgG antibodies against p54 and F317L in both the G1 and G2 groups gradually increased from 0 to 28 dpi, serum IgG antibody in the G1 group increased significantly at 28 dpi compared to that at 0 dpi (p54 for *p <*0.05, and F317L for *p <*0.01) and 7 dpi (F317L, *p <*0.01), and serum IgG antibody against p54 at 14 dpi and 28 dpi in G2 was significantly higher than that at 0 dpi (*p <*0.01) and 14 dpi (*p <*0.05). The serum IgG antibody against p54 protein in the G2 group was significantly higher than that in the G1 group at 28 dpi (*p <*0.01). For the B602L protein, the secretion of serum IgG antibody in G1 and G2 groups increased rapidly in the period of 0 to 28 dpi, and the secretion in G1 increased fastest in the period of 14 to 28 dpi, with extremely significant differences between those at dpi 28 and dpi 14 (*p <*0.001) and dpi 28 and dpi 0 (*p <*0.001), while that of serum IgG antibody in the G2 group increased fastest in the period of 7 to 14 dpi, with significant differences between those at dpi 28 and dpi 14 (*p <*0.01) and dpi 14 and dpi 0 (*p <*0.001).

The ASFV whole virus protein was also used to detect IgG antibodies in the serum of animals immunized at 0, 7, 14 and 28 dpi. As shown in **Figure 7B**, the serum IgG antibody secretion of the G1 and G2 groups increased rapidly within 0 to 7 dpi, which was significantly higher than that at 0 dpi (*p <*0.001). Although the antibody level decreased slightly from 7 to 28 dpi, there was still a significant difference compared with that at 0 dpi (*p <*0.05, *p <*0.001).

The p22 plus p30 protein coating was used to detect the sIgA antibody in the oral fluid of animals immunized at 0, 7, 14 and 28 dpi. As shown in **Figure 7C**, sIgA antibody in G1 and G2 groups increased rapidly in the period of 0 to 28 dpi, the secretion of oral fluid sIgA antibody in G1 and G2 at 28 dpi was significantly higher than that at 0 dpi (*p <*0.001), and the secretion of sIgA antibody at 14 dpi in G2 was significantly higher than that at 7 dpi (*p <*0.01). Overall, the secretion of sIgA antibody in the oral fluid of the G2 group was higher than that of the G1 group, but there was no significant difference.

### 3.9 Related pig serum cytokine secretion levels increased after immunization using both real-time PCR and ELISA method

**Figure 8:**
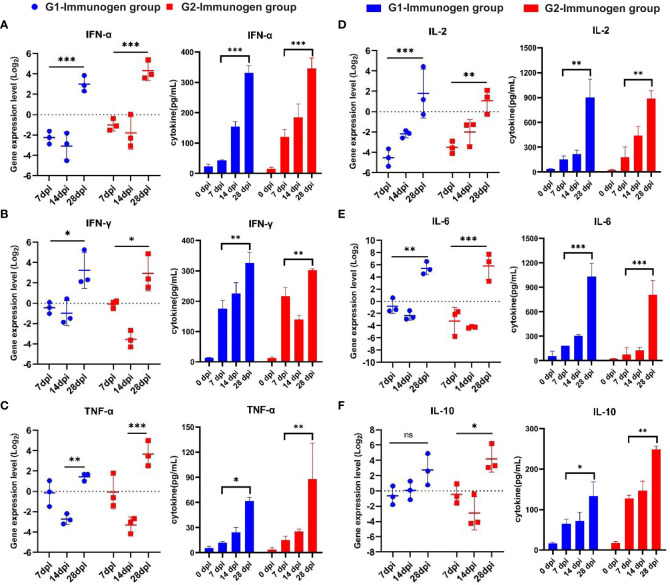
Serum cytokine expression level detection from candidate immunogen-immunized pigs with the method of real-time PCR and using related ELISA detection kits. The relative fluorescence quantitative PCR method was used to detect the secretion level of cytokine including IL-2, IL-6, TNF-α, IL-10, IFN-α, IFN-γ mRNA in serum of pigs in immune group and control group, and the results were analyzed by method 2^^-ΔΔCt^. Relative cytokine expression levels induced by two combination of candidate immunogens compared to pigs immunized with PBS (set to 1). For ELISA method to test secretion of serum cytokines, according to the standard curve drawn by each cytokine standard curve, concentrations of the above six cytokines from each pig serum of two combination of candidate immunogens were calculated according to the (OD450-OD630) value. **(A)** IFN-α; **(B)** IL-2; **(C)** IFN-γ; **(D)** IL-6; **(E)** TNF-α; **(F)** IL-10; *: *p* < 0.05, represents the difference is significant, **: *p* < 0.01, ***: *p* < 0.001, represents the difference is extremely significant.

As shown in **Figure 8A**, the interferon-α (IFN-α) expression level from serum in both G1 and G2 groups were downregulated at 7 and 14 dpi, and the secretion at 28 dpi was significantly higher than that at 7 dpi (*p <*0.001). Although the expression of IFN-α in the G2 group was slightly higher than that in the G1 group at 28 dpi, there was no significant difference. The expression of the interferon-γ (IFN-γ) in G1 and G2 groups was downregulated at 14 dpi; similarly, the secretion of IFN-γ at 28 dpi was significantly higher than that at 7 dpi (*p <*0.05, **Figure 8C**), and the expression levels remained basically the same at 28 dpi in both experimental groups. The expression of tumor necrosis factor-α (TNF-α) in G1 and G2 groups was downregulated at 14 dpi, and TNF-α secretion increased significantly at 28 dpi in both the G1 (*p <*0.01, **Figure 8E**) and G2 groups (*p <*0.001, **Figure 8E**).

Similar to IFN-α, at 28 dpi, the expression of G1 was lower than that in the G2 group, but with no significant difference. The expression of interleukin-2 (IL-2) (**Figure 8B**) and interleukin-6 (IL-6) (**Figure 8D**) in Groups G1 and G2 was downregulated at 7 dpi and 14 dpi. Similar to TNF-α, the secretion of IL-2 and IL-6 at 28 dpi was significantly higher than that at 7 dpi in both the G1 (*p <*0.01, **Figure 8B**; *p <*0.001, **Figure 8D**) and G2 (*p <*0.01, **Figure 8B**; *p <*0.001, **Figure 8D**) groups. At 28 dpi, IL-2 expression in the G1 group was higher than that in the G2 group, but the difference was not significant (**Figure 8B**). Although the expression of IL-6 in the G2 group was slightly higher than that in the G1 group at 28 dpi, there was no significant difference (**Figure 8D**). For interleukin-10 (IL-10), as shown in **Figure 8F**, although the secretion in the G1 group at 28 dpi was higher than that at 0 dpi, with no significant difference. The expression of IL-10 in the G2 group was downregulated at 14 dpi, and the secretion was significantly higher at 28 dpi than at 7 dpi (*p <*0.05, **Figure 8F**). At 28 dpi, the expression of G1 was lower than that in the G2 group, but there was no significant difference.

The results of serum cytokine concentration detected by ELISA method were consistent with those via method of RT-PCR, only the significant differences of the other five cytokines at specific time points are different except for IFN-α and the secretion of cytokines in immune group reached peak at 28 dpi.

## 4 Discussion

ASFV is a large and complex DNA virus that encodes approximately 150 proteins (23). The large and complex structure of the virus makes the development of an ASFV vaccine difficult. Unlike virus-based vaccines, subunit vaccines are able to provide a safer immunization strategy (15). Previous studies of experimental subunit vaccines based on several antigens produced different protective effects, suggesting that these antigens do play some role in host protection (24). However, this result was not stable, and partly baculovirus-based expression of proteins, although inducing neutralizing antibodies and delaying the onset of clinical signs, ultimately did not protect animals from severe disease (14). Thus, screening and identification of antigens capable of inducing protective responses by appropriate means, together with the use of appropriate antigen delivery pathways and adjuvants (4).

In this study, immunoblotting experiments with ASF convalescent pig serum and ASFV whole virus proteins were performed. After identification by qualitative mass spectrometry identification of proteins, combined with the study conducted in our lab by screening MHC-II presenting peptides in ASFV-positive pig urine (data not shown) and related literature reports (16), seven candidate immunogens were finally determined, and purification of recombinant proteins by both prokaryotic *E. coli* and baculovirus expression systems was performed. Previous studies have identified the principal serological immunodeterminants of ASFV by screening a virus cDNA library with antibody (25), 14 potential immunodeterminants were identified, three of them are B602L, p54 and p10, which were also three of the seven antigen candidates selected in this study. Thus, using ASFV-convalescent, ASFV-positive pig serum and ASFV-negative pig serum hybridized with the whole virus protein, to screen out the candidate immunogens with potential protection is possible.

Although p72 protein is the largest structural protein ASFV, and here it was detected by mass spectrometry, however, previous studies have proved that p72 protein was widely used in pathogen detection and had no obvious immune protection (14, 15). Therefore, p72 was not selected as a candidate immunogens in this study. In the subsequent protein hybridization identification of pig-positive and convalescent serum as primary antibodies, p22 protein can induce a strong reaction with both ASFV-convalescent and ASFV-positive pig serum; however, the p10 protein could not react with ASFV-convalescent pig serum. In addition, the F317L protein showed a binding reaction with ASFV-positive pig serum, related studies have demonstrated that F317L interacts with IKKβ, which leads to the production of inflammatory cytokines and an impaired antiviral response. Unexpectedly, the F317L protein also seems to play a crucial role in the replication of ASFV (26), which could partially explain why the F317L protein can react with ASFV-positive serum but cannot be detected against ASFV-convalescent serum because the inflammatory and antiviral responses produced by the F317L protein are transient. Polyclonal antibody against the p22 protein reacted well with the p22 protein and whole virus protein; however, the p10 protein could not bind specifically with the whole virus protein, which may be because p10 plays an important role in the nucleus in the later period of the replication cycle (27, 28), suggesting that p10 is a marker protein candidate for the early diagnosis of ASFV, which was consistent with our recent study (29) that recombinant p10 protein could not react with the serum of convalescent pigs but could react with positive pig serum.

Humoral immunity is an important part of the immune system, and antibodies can reduce virus infectivity by attaching to virus particles. IgG antibody levels were greatly elevated after immunization was strengthened. In general, the level of antigen-specific IgG secretion in the G1 group was higher than that in G2 group. However, serum IgG antibody secretion against the whole virus in the two groups remained almost the same. In addition, an obvious increase in mucosal antibody expression levels appeared after enhanced immunization. A previous study confirmed that p30 showed the earliest sIgA-positive conversion in oral fluid (29). Here, sIgA antibody detection used p22 plus p30 as the double coating antigen, as p30 and p22 are antigens that exhibit significant responses in the early and late phases of ASFV infection, and the sensitivity and specificity of this double-coated assay were better than those using p30 antigen only (data not shown). In addition, MONTANIDETM1313 VG N mucosal adjuvant, which can stimulate strong mucosal immunity, was used in the G1 group. Consistent with our previous research (19), mucosal and cellular immune responses were elicited by nasal and intramuscular inoculation, but the overall trend was weaker than that in the G2 group, which may be due to the expression mode of the immunogen, the choice of adjuvant and species differences between mice and pigs. In addition to activating cellular immunity, Montanide ISA 51 VG adjuvants can lead to a long-term immune response by slowing the release of antigens (30). Previous studies have also confirmed that p30 and p54 could activate the specific mucosal immunity for controlling ASFV infection (31). Therefore, to better validate the mucosal immunity, p10, p30, and F317L proteins were added as candidate immunogens in Immunogen-G1, to conduct comparison with Immunogen-G2.

Lots of previous studies have shown that cellular immune response contributes greatly to the protective immunity of host against ASFV infection (32–36). Interferon gamma (IFN-γ) is primarily secreted by CD4^+^ Th1 cells, NK cells, and CD8^+^ cytotoxic T cells and plays a pivotal role in inducing and modulating an array of immune responses (37). IFN-γ^+^ lymphocytes from ASFV-stimulated immune PBMCs displayed mainly the CD4^+^CD8^+^ T-cell phenotype (38). Anti-CD4 and CD8 monoclonal antibodies could also block the specific proliferation of ASFV under the stimulation of infectious virus, while under the stimulation of ultraviolet inactivated virus, anti-CD8 monoclonal antibodies only inhibit the specific proliferation of ASFV by 60%, but anti-CD4 mAb could completely block it (39), and a strong ASFV-specific IFN-γ response and immunostaining of CD4^+^ T cells in the spleens of vaccinated pigs (40). Here, the CD4^+^/CD3^+^ T cells in the two immune groups increased significantly, and the upward trend in the Immunogen Group 1 was slightly stronger. At the same time, consistent with previous studies (41), the ratio of CD4^+^/CD8^+^ T cells was significantly higher than that of the control group. Although previous investigation showed that CD8^+^ T cells play a crucial role in ASFV protection (33), here, neither of the two immunogen immunization groups in this study activated CD8^+^ T cells. More research will be carried out on why this phenomenon occurs in future studies.

Acting as a pleiotropic cytokine, IL-2 is released by activating CD4^+^ and CD8^+^ T cells, which could play a synergistic role with IFN-γ to promote the Th1 cell response jointly (42). In addition, IL-2 and IFN-γ could promote the proliferation and activation of NK cells to form CHAK cells (CC-chemokine-activated killer) (43). In this study, both IL-2 and IFN-γ expression levels in immunized pig serum were upregulated after enhanced immunization in both G1 and G2 immune groups. T-regulatory cells (T-regs) can inhibit the secretion of proinflammatory Th1 cytokines(44), but the expression level of IL-10, as one of the markers, is not obvious, suggesting that after immunization, host can be stimulated to secrete related cytokines, thus promoting the Th1 cell immune response. A previous study showed that an increase in IL-10 serum levels was positively correlated with the survival of pigs infected with the moderately virulent isolate Netherland’86 (45). In addition, IFN-α levels peaked early after ASFV infection (1-3 dpi) (46, 47), and the infection of pigs with the attenuated deletion mutant ASFV-Δ7R can result in increased serum levels of IFN-α (48). Interestingly, IFN-α can increase the level of perforin, which leads to the enhancement of NK activity, which in turn can also promote the differentiation of Th1 cells (49), which was consistent with upregulated expression of IL-10 and IFN-α from serum in this study. As proinflammatory cytokines, IL-6 and TNF-α have been proven to be secreted in large quantities in the body infected with ASFV and induce apoptosis of porcine lymphocytes (50). Here, the gene expression levels of IL-6 and TNF-α in the immune groups were also upregulated, which may also be related to the immune response mediated by Th1 cells.

In conclusion, seven candidate immunogens were screened and selected based on ASFV-positive and ASFV-convalescent sera against whole ASFV virus, combined with proteins corresponding to MHC-II presenting peptides screened from ASFV-positive pig urine and related literature reports. Two immunogen immunization groups were prepared by prokaryotic and eukaryotic protein expression. After immunizing animals, two immunogen groups with different combinations and immunization methods can stimulate host to produce ideal immune responses, including humoral, mucosal and cellular immune responses, which laid a theoretical foundation for the follow-up research and immune protection test of two combination of candidate immunogens

## Data availability statement

The original contributions presented in the study are included in the article/supplementary material. Further inquiries can be directed to the corresponding authors.

## Ethics statement

The animal study was approved by the Committee on the Ethics of Animal Experiments (Protocol # PDC 2022013) and were performed in strict accordance with the animal regulations of Jiangsu Province (Government Decree No. 45) at Jiangsu Academy of Agricultural Sciences (License No. SYXK (Su) 2020-0023). The study was conducted in accordance with the local legislation and institutional requirements.

## Author contributions

LX, FH and and DJ conducted most of the experiments and wrote the manuscript. RC helped with protein expression and purification of prokaryotic expression. YG and LZ helped sample collection, animal immunization. MY and J-WL helped with protein expression and purification. YY and YB helped with flow cytometry analysis and oral fluid sIgA antibody detection. ZZ provided ASFV-convalescent pig serum and helped with mass spectrometry identification and analysis. YL, QX, GS and YW helped with cytokine expression level analysis and mass spectrometry identification. ZF, DS and XX conceived this study and contributed in the design as well as coordination. All authors contributed to the article and approved the submitted version.
